# Progress and Perspectives of Mid-Infrared Photoacoustic Spectroscopy for Non-Invasive Glucose Detection

**DOI:** 10.3390/bios13070716

**Published:** 2023-07-07

**Authors:** Md Rejvi Kaysir, Jiaqi Song, Shazzad Rassel, Abdulrahman Aloraynan, Dayan Ban

**Affiliations:** 1 Department of Electrical and Computer Engineering, University of Waterloo, 200 University Ave. W, Waterloo, ON N2L 3G1, Canada; 2 Waterloo Institute for Nanotechnology, University of Waterloo, 200 University Ave. W, Waterloo, ON N2L 3G1, Canada; 3 Department of Electrical and Electronic Engineering, Khulna University of Engineering & Technology, Khulna 9203, Bangladesh; 4 Department of Physics and Astronomy, University of Waterloo, 200 University Ave. W, Waterloo, ON N2L 3G1, Canada

**Keywords:** diabetes, non-invasive glucose detection, photoacoustic spectroscopy, mid-infrared spectrum, machine learning, quantum cascade laser

## Abstract

The prevalence of diabetes is rapidly increasing worldwide and can lead to a range of severe health complications that have the potential to be life-threatening. Patients need to monitor and control blood glucose levels as it has no cure. The development of non-invasive techniques for the measurement of blood glucose based on photoacoustic spectroscopy (PAS) has advanced tremendously in the last couple of years. Among them, PAS in the mid-infrared (MIR) region shows great promise as it shows the distinct fingerprint region for glucose. However, two problems are generally encountered when it is applied to monitor real samples for in vivo measurements in this MIR spectral range: (i) low penetration depth of MIR light into the human skin, and (ii) the effect of other interfering components in blood, which affects the selectivity of the detection system. This review paper systematically describes the basics of PAS in the MIR region, along with recent developments, technical challenges, and data analysis strategies, and proposes improvements for the detection sensitivity of glucose concentration in human bodies. It also highlights the recent trends of incorporating machine learning (ML) to enhance the detection sensitivity of the overall system. With further optimization of the experimental setup and incorporation of ML, this PAS in the MIR spectral region could be a viable solution for the non-invasive measurement of blood glucose in the near future.

## 1. Introduction

Diabetes mellitus (DM), which is commonly referred to as diabetes, is a prevalent and enduring/chronic ailment in the contemporary world. It is a disorder in which the body cannot produce enough (type-1) or respond normally (type-2) to insulin, causing blood sugar levels to be abnormally high. Table 1 provides an overview of the customary levels of glucose in the blood of adults in different conditions found with different testing methods. As per the diabetes report published by the World Health Organization, the number of individuals affected by diabetes across the world increased from 108 million in 1980 to 422 million in 2014 [1]. In addition, DM was directly responsible for causing 1.5 million deaths in 2019. Although there is currently no cure for diabetes, it can be managed through monitoring and regulating blood sugar levels.

Therefore, individuals with diabetes must check their blood sugar levels multiple times a day. The traditional methods of measuring blood sugar levels involve invasive enzyme reaction-based procedures that entail pricking fingertips to draw blood, which can be an unpleasant and painful experience. In addition, the compromised immune systems of individuals with diabetes make them susceptible to infections caused by needle pricks in various areas of the body [3]. Therefore, there is an urgent demand for the development of a new non-invasive method for glucose monitoring that could give an accuracy comparable to commercial invasive devices.

It is estimated that the global market for blood glucose monitoring devices will grow from its value of around USD 14.48 billion in 2021 to USD 28.75 billion by 2030 [4]. Numerous researchers and companies have endeavored to create non-invasive glucose monitoring devices in pursuit of this promising concept of measuring blood glucose levels without the need to prick fingers. The latest technological advancements in this field incorporate a variety of optical [5,6] and non-optical [7,8] methods. In another sense, non-invasive glucose monitoring systems can also be classified into three categories: EM wave, transdermal, and enzymatic. A brief description of these technologies with relative advantages and disadvantages is found in Ref. [9]. In addition, the details of some other different non-invasive techniques for glucose sensing can be found in recent review works [8,10,11]. Nonetheless, none of these methods have been able to satisfy the physiological demands due to inadequate precision and operational instability [12]. Consequently, the demand for developing better methods of glucose monitoring is necessary.

**Figure 1:**
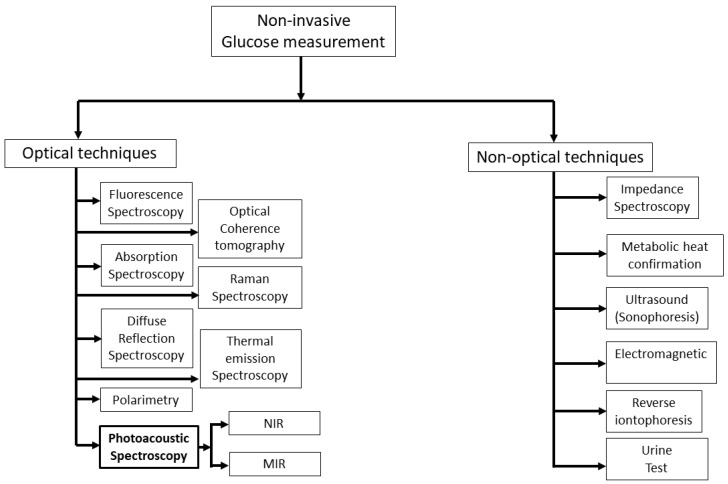
Summary of prospective active research areas in techniques for measuring glucose levels non-invasively. NIR: near-infrared; MIR: mid-infrared.

Figure 1 shows the overview of a vast area of non-invasive glucose measurement techniques that are currently active areas of research. For several years, researchers have been striving to devise a non-invasive method for detecting glucose levels in the body without the need for pricking. This pursuit has led to the exploration of various methods, which can be broadly categorized into two types: optical and non-optical approaches.

In general, non-optical spectroscopy measures the non-optical properties of human skin. There are several non-optical approaches in the literature such as impedance spectroscopy [7], electromagnetic [13], reverse iontophoresis [14], metabolic heat confirmation [15], and ultrasonic [16]. Among them, impedance spectroscopy has shown great promise in recent years as it measures glucose levels directly, rather than relying on indirect measures such as blood oxygen levels or skin temperature. Moreover, it provides real-time glucose monitoring, which means that it can measure glucose levels continuously and provide immediate feedback. Nonetheless, this approach has the potential to alter the properties of the skin and induce blistering, irritation, or erythema [7]. In addition, this method also requires precise instrumentation and careful calibration, which can be time-consuming and difficult to perform.

Optical spectroscopy is less susceptible to skin irritation in comparison to non-optical methods, and it exhibits a high level of specificity for detecting glucose, even in a complex matrix such as blood. There are different optical techniques, such as fluorescence spectroscopy [17,18], optical coherence tomography [19], Raman spectroscopy [20,21], thermal emission spectroscopy [22,23], diffuse reflectance spectroscopy [24,25], polarimetry [26,27], absorption spectroscopy [28,29], and photoacoustic spectroscopy [30,31], that have been used to sense glucose non-invasively. In addition, FTIR (Fourier Transform Infrared) spectroscopy is a widely used analytical technique for measuring the absorbance of a sample by infrared (IR) radiation. An FTIR spectrometer consists of a broadband IR light source (e.g., halogen lamp), an interferometer, a sample compartment, and a detector. The IR light source in the FTIR spectrometer is weak and it is very difficult to obtain even transmission spectra throughout the experiment. Recently, Chen et al. proposed a modified FTIR method where they obtained transmission spectra of glucose at different concentrations with a correlation factor over 99% by using a strong quantum cascade laser (QCL) with 600 mW of output power [32].

In recent years, PAS has been proven effective in detecting glucose non-invasively because it provides greater sensitivity compared to optical absorption spectroscopy [33,34,35]. In this approach, a modulated laser beam in the IR range is directed onto the skin, causing thermal expansion. This expansion creates an acoustic wave that is influenced by both the absorption coefficient of the sample and the physical characteristics of the propagation medium, such as the thermal expansion coefficient and acoustic velocity [36,37]. Generally, PAS is utilized to detect the vibration modes of glucose molecules at a specific excitation wavelength, where considerable absorption occurs. The main advantage of acoustic signals is that it shows less interference in biological samples than any optical signals. Additionally, an acoustic microphone is utilized as a detector to pick up the signals, resulting in a significant reduction in the cost of the detector.

PAS can be classified based on the wavelength range of the excitation source: near-infrared (NIR), and mid-infrared (MIR) wavelength range. This is due to the glucose-specific absorption of chemicals in that region. Most of the attempts to develop non-invasive glucose monitoring devices employ NIR light, as it can penetrate several millimeters into human tissue. However, glucose absorption within this range is weak and significantly interferes with other blood and tissue components [38,39], making it difficult to effectively detect blood glucose non-invasively. On the other hand, glucose shows a strong absorption signature in the MIR region with less interference with other tissue components [40]. One of the significant challenges in this field is the limited penetration depth of mid-infrared light below 100 µm into human skin due to strong water absorption [41,42]. Therefore, it is possible to detect glucose within the interstitial fluid (ISF) of the epidermis as the blood capillaries are not reached. Clinical trials have confirmed the strong correlation between blood glucose levels and the concentration of glucose in the ISF. This is because metabolites and proteins diffuse into the ISF from the blood capillaries as they make their way to the cells [43]. Laser sources operating in the MIR range have the benefit of producing more robust photoacoustic signals and exhibiting measurement stability.

In this article, we aimed to give an overview of the chronological development of MIR-based PAS for non-invasive glucose sensing, where we focused on two parts: (i) technical/experimental progress and challenges, and (ii) data analysis for correlations with glucose. As the first concept of incorporating an MIR light source in PAS for non-invasive glucose sensing was first implemented in 2005, this review focused on the development and progress of this technology to date. This work is presented in the following manner: Section 2 illustrates the basic principles and underlying physics behind the conventional PAS. It also focuses on the advantages of using MIR light as the excitation source. In Section 3, the chronological development of MIR-based PAS is summarized, and various previously published extensive experimental results are presented with proper explanation. We also focused on the experimental/instrumental challenges of MIR-based PAS for non-invasive blood glucose detection. Section 4 focuses on the recent trend of introducing Machine Learning (ML) in PAS to enhance the detection sensitivity of non-invasive blood glucose detection. Finally, some prospects for MIR-based PAS are discussed in Section 5.

## 2. Basics of Photoacoustic Spectroscopy for Glucose Sensing

Although the photoacoustic effect was discovered by Bell in 1880, Viengerov’s 1938 publication marked the introduction of using PAS for gas concentration detection [44]. This paper highlights the potential application of PAS in material characterization. However, due to limitations in light sources, the progress of PAS was slow, and there were no significant advancements in subsequent decades. In the 1970s, the invention of lasers and advancements in acoustic sensor technology revitalized PAS. Notably, Kreuzer’s experiment using a laser for Photoacoustic gas detection [45] rekindled interest among researchers, establishing PAS as one of the most promising technologies in the fields of biology and medicine. Now, PAS is considered to be a highly sensitive method that can be used for chemical sensing, bio-sensing, and bio-imaging, utilizing an infrared laser as the light source. As the penetration depth of MIR light into the biological sample is small (< 100 µm) compared to visible and NIR light due to the high absorption coefficient of background water, excitation light in the MIR region can only reach the epidermis layer, generating acoustic waves and obtaining glucose information from the ISF under the skin. Therefore, by combining PAS with an MIR light source, it is possible to detect blood glucose levels non-invasively from the human skin.

In the following sections, the basic principle of generating an acoustic signal from a human skin sample using a modulated laser beam and the factors affecting the acoustic signal are described. They also highlight the basic requirements of the instrumentation needed for MIR-based PAS. Furthermore, the detailed advantages of using MIR light as an excitation source for PAS are also discussed.

### 2.1. Generation and Behavior of an Acoustic Signal Using MIR Light Source

**Figure 2:**
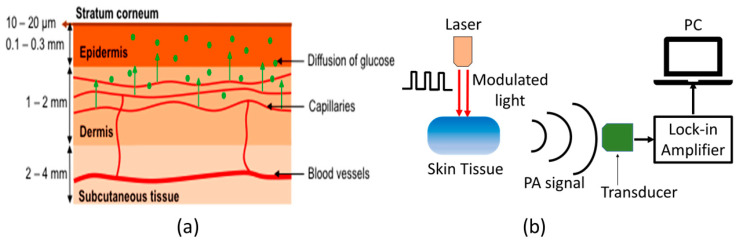
(**a**) Schematic representation of the components of human skin [46], and (**b**) the basic principle of generation and detection of acoustic signal for PAS. Reprinted with permission from Ref. [46]. 2016, MDPI.

Figure 2a shows the schematic of typical human skin, which consists of mainly three layers: epidermis, dermis, and subcutaneous tissue [46]. In general, MIR-based PAS utilizes modulated MIR laser light that can penetrate through the human tissue and reach the ISF of the epidermal layer, where acoustic signals are generated by the absorption of glucose molecules. The MIR light is strongly absorbed by glucose molecules between 8–10 µm wavelength due to the fundamental vibrational resonances [47,48]. The generated acoustic signals can travel through the human tissue with minimal scattering and be detected by a microphone. Thus, the glucose information in ISF can be correlated to the acoustic signal in MIR-based PAS. The ISF is a layer of biological fluid found between cells and consists of water, solvents, and blood vessels. It contains various substances such as sugars, fats, amino acids, hormones, coenzymes, white blood cells, and cellular waste products. Glucose is known to diffuse from the blood to the ISF layers, with a delay of 5 to 15 min [49,50].

Figure 2b shows the basic experimental setup for generating and detecting the photoacoustic signal. The principle of PAS involves the generation of acoustic waves through a pulsed electromagnetic source. These electromagnetic waves are absorbed by a biological object, causing thermal expansion or pressure, resulting in the production of acoustic waves [51,52]. Sensitive ultrasonic or piezoelectric sensors can then detect these waves, which can be distinguished between different materials. Additionally, the characteristics of the modulated light source, including the modulation amplitude and duty cycle, can significantly affect the generation of acoustic waves. The properties of human skin are also crucial to consider when generating these waves.

The pressure produced by the photoacoustic effect can be expressed by the following wave equation with spatial heat distribution H (*r*, *t*), according to the laws of thermodynamics [53]:

(1) $$\nabla^{2}p\left(r,t\right)-\frac{1}{v^{2}}\frac{\delta^{2}}{\delta t^{2}}p\left(r,t\right)=-\frac{\beta }{C_{p}}\frac{\delta }{\delta t}H(r,t)$$

where, $p\left(r,t\right)$ represents spatial pressure distribution at time *t*, *v* is the acoustic velocity, *Cp* is the heat capacity, and β is the thermal expansion coefficient.

The equation below can be used to represent the peak pressure (*P*) for a medium that absorbs light weakly:

(2) $$P=k\frac{\beta v^{n}}{C_{p}}E_{0}\alpha$$

Here, *k* is the system constant, *E*_0_ is the incident laser pulse energy, and *n* is the constant between 1 and 2, depending on the experimental condition. Sometimes, the combination of experimental parameters can lead to a stronger photoacoustic signal compared to the traditional spectroscopic method. It should be noted that the response from glucose does not give a sufficient amount of signal enhancement for the detection, which requires a photoacoustic cell (PAC) for signal amplification.

### 2.2. Required Instrumentation for MIR-Based PAS

As shown in Figure 2b, the MIR-based PAS experimental setup consists of mainly four parts: MIR light source, acoustic resonator/PAC, acoustic detector, and lock-in amplifier. New advancements in technology for generating mid-IR light have emerged, particularly in the form of pulsed QCLs. These lasers are capable of delivering high peak powers, reaching hundreds of milliwatts, while still maintaining average powers in the range of a few milliwatts [54]. As a result, it is now possible to obtain stronger signals from areas of the skin that were previously thought to be incapable of detecting MIR light [55]. In general, the generated photoacoustic signal is weak and needs to be amplified. This amplification is done by exciting the corresponding acoustic mode in an acoustic resonator [56]. This helps to significantly enhance the generated photoacoustic signal, and thus, enhance the overall detection sensitivity of PAS. In general, a resonator with an open-ended shape resembling the letter “T” is employed to minimize the buildup of moisture inside the enclosure, while also mitigating variations in air temperature and pressure that occur during measurements [56,57]. To convert the amplified acoustic signal from the PAC, a sensitive microphone [35] or piezoelectric [58] sensor is used. The electrical signal from the sensor is amplified by lock-in amplifier (LIA), which can be further processed by using the computer. It is noted that different sensors are employed in the PAS setup to maintain different environmental factors (such as temperature, pressure, and humidity) constant throughout the experiment.

By optimizing the design of the PAC and choosing an appropriate modulation frequency, the acoustic signals can be improved, leading to increased detection sensitivity. The system is designed to detect changes in acoustic signals resulting from variations in the absorption coefficient of glucose solutions. As the concentration of glucose in the solution increases, the absorption coefficient also increases, which in turn generates higher acoustic signals.

### 2.3. Spectral Response of Glucose in the MIR Region

**Figure 3:**
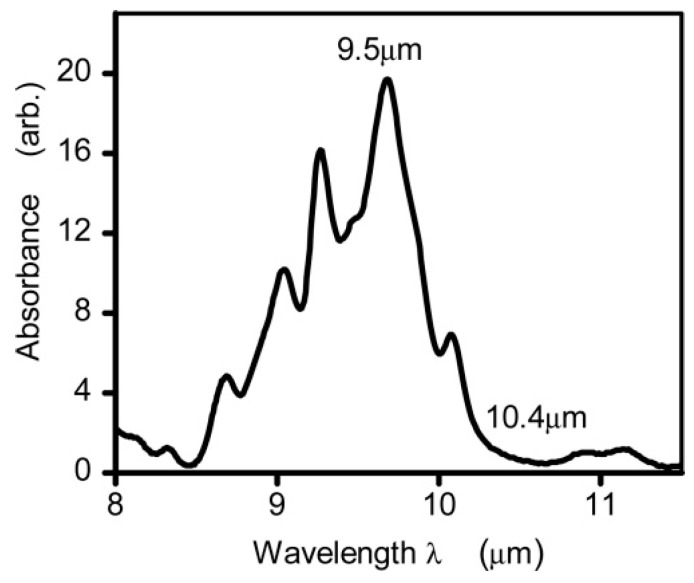
The absorption spectrum of glucose in the mid-infrared range was measured using Fourier Transform Infrared spectroscopy. The measurements were taken from glucose solutions in water, with the absorption of water serving as a reference and removed from the readings [59]. Reprinted with permission from Ref. [59]. 2015, The Optical Society.

Unlike NIR light, glucose exhibits significant absorption characteristics in the infrared spectrum due to the stretching and bending vibration of its C–H–O bonds within the 800–1200 cm^−1^ (i.e., 8.3–12.5 µm) range [50]. Figure 3 shows the glucose absorption spectra in the MIR region, where several absorption peaks are observed [59]. The highest and lowest peaks are found to be at 9.5 µm and 10.4 µm, respectively. However, it is important to note that the absorption coefficient of blood glucose is in the range of ~ 10^−3^ cm^−1^, which can vary depending on other factors/components in blood samples. As stated, MIR-based PAS collects the information from ISF, which serves as a significantly transparent medium compared to blood, primarily composed of glucose, albumin, and small amounts of lactate [35]. The main advantages of using the MIR spectrum for glucose detection are given below:

(i) Greater absorption: Glucose has a relatively weak absorption in the visible and NIR regions, but it has strong absorption in the MIR region. That means the sensitivity of glucose detection in the MIR region can be enhanced.
(ii) High signal-to-noise (from water) ratio: Water has strong absorption in the MIR region in comparison to visible and NIR regions, which can interfere with the detection of glucose. However, as glucose has distinct absorption in the MIR region, the overall signal-to-noise ratio can be improved by selecting an excitation wavelength close to the peak absorption wavelength in the MIR region.
(iii) Ability to detect glucose in complex matrices: The use of MIR PAS can improve the detection of glucose in complex biological matrices, such as blood, by avoiding the interference caused by other molecules present in the matrices.
(iv) Greater specificity: MIR-based PAS can provide greater specificity for glucose detection as it has distinct absorption characteristics in the MIR wavelength region compared to visible and NIR wavelength regions. Thus, MIR-based PAS utilizing multiple wavelengths in the MIR region can be used significantly to improve the specificity of glucose sensing. It should be noted that specificity can be improved by multiple wavelengths in other wavelength regions, where glucose has a good absorption coefficient.

## 3. Progress of MIR-Based PAS in Glucose Sensing

### 3.1. Summary of the Chronological Development

**Figure 4:**
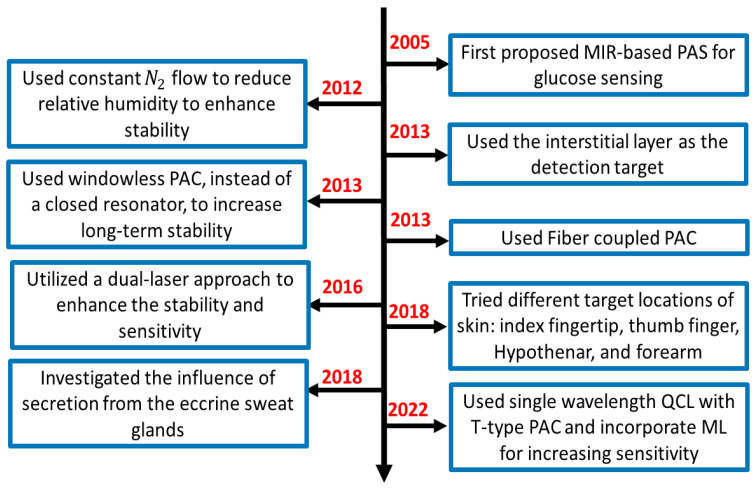
The flow chart of the summary of the recent development of MIR-based PAS for non-invasive glucose sensing. PAC: photoacoustic cell.

This section provides a summary of the recent development of MIR-based PAS, basic experimental setup, data analysis strategies, and possible challenges that need to be overcome for the practical implementation of this technology into the potential future market. Figure 4 shows the flowchart of the recent approaches and development of PAS in the MIR region for non-invasive glucose sensing. It is shown that MIR-based PAS was first implemented in 2005, where the researchers utilized MIR lasers as the excitation sources to generate the photoacoustic signal, which could enhance the sensitivity and stability of the system. However, the background water in a glucose solution and other environmental parameters were core issues that greatly affected the accuracy of glucose detection. To eliminate the effect of such background noises, several strategies were taken to reduce the effect of environmental conditions by different research groups.

In general, the experimental setup of PAS incorporates a PAC to amplify the acoustic signal to be detected by lock-in amplifier (LIA). In 2012, some researchers used nitrogen (N_2_) gas to reduce the relative humidity in a PAC. Later, in 2013, they utilized the windowless PAC to control the relative humidity at a low level, which enabled long-term stability. Meanwhile, many techniques were used to enhance the accuracy of the system. In 2013, a fiber-coupled PA cell was used to make the sensor more compact and convenient for in-vivo measurement. In addition, the detection location of the skin was also taken into consideration. Due to the characteristic of MIR light, the penetration depth is below 100 μm, which cannot reach the blood vessels or capillaries. Instead, they reach the epidermis layer, which contains the ISF. Then it becomes an important detection target for in vivo detection. In 2016, the same research group utilized two laser approaches to further enhance the non-invasive glucose detection sensitivity and long-term stability.

Later, some researchers tried to find the optimal locations on the skin in 2018; in the same year, another research group designed an experiment to find out the influence of secretion from the eccrine sweat glands and to find the reasons and solutions for the fluctuation in the spectrum using microscopic PAS. Most recently, Aloraynan et al. used a single wavelength laser that has the second strongest absorption of glucose in the MIR region (see Figure 3), where they achieved good prediction accuracy by introducing machine learning for a given set of photoacoustic signal data.

In summary, in the development of the MIR-based PAS, researchers dedicated themselves to reducing the impact of the diversified environment, such as relative humidity, temperature, and pressure. Moreover, in the mid-infrared region, water absorption is a key issue that needs to be effectively addressed. Then, the detection positions on the skin also play an important role in the stability and accuracy of prediction. The general details of the recent articles are summarized in the following Table 2.

### 3.2. Experimental/Instrumentation Development and Data Analysis

**Figure 5:**
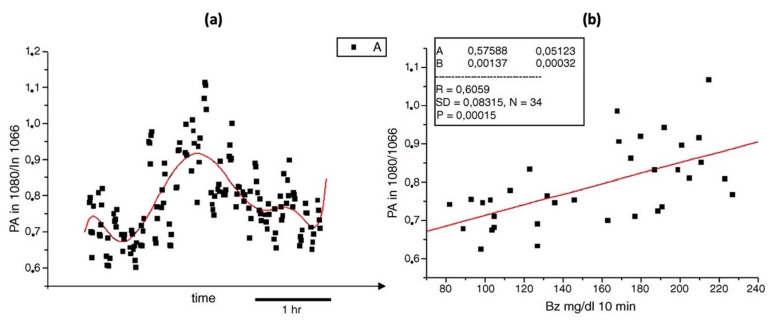
(**a**) Photoacoustic signals (black points) taken during oral glucose tolerance test (red solid line). (**b**) The correlation between the average photoacoustic signals and the corresponding blood glucose values obtained during an oral glucose tolerance test [60]. Reprinted with permission from Ref. [60]. 2005, Elsevier.

As stated in the previous Section, the combination of MIR lasers and PAS was first introduced in 2005. This method overcame the difficulties of other methods suffered by low sensitivity or low intensity of output signal at that time. It can successfully correlate the photoacoustic signal and the blood glucose level in an in vivo environment, which can lead to possible non-invasive glucose detection in the future. However, due to the technique being immature at that time, it did not consider the physical environment impact, such as humidity or temperature, which can greatly influence the experimental results. As a result, a poor correlation was obtained, which is shown in Figure 5.

**Figure 6:**
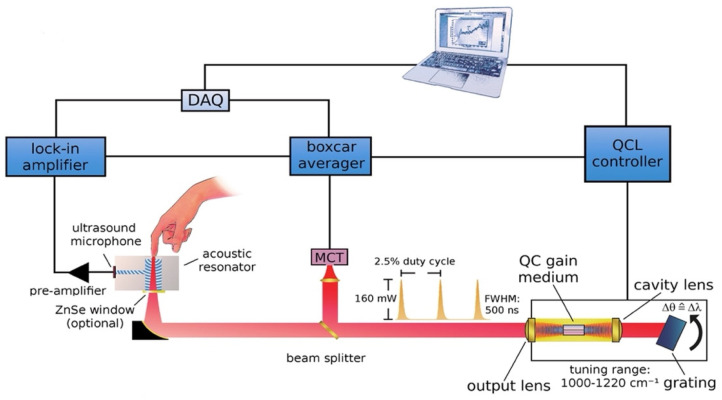
Schematic depicting the configuration of the experimental arrangement for the measurement of glucose in a non-invasive manner using MIR-based PAS [35]. Reprinted with permission from Ref. [35]. 2013, American Chemical Society.

Later, this new technique was further developed and improved. The same research group that introduced the combination of the MIR and PAS delved deeper into the detection target in the ISF of human skin. Figure 6 displays the upgraded experimental arrangement, wherein a tunable external cavity QCL is directed towards a PAC and focused on the sample. The external cavity laser has the ability to tune within a range of 1000 to 1220 cm^−1^ and can produce a maximum pulse output power of 160 mW at 1170 cm^−1^.

**Figure 7:**
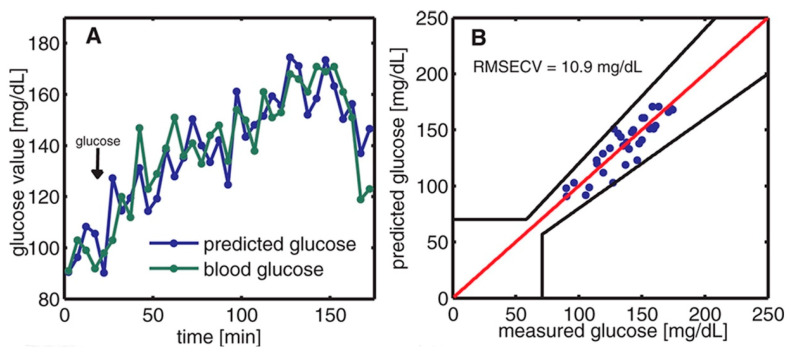
(**A**) The time variation of blood glucose levels was monitored through two methods: enzymatic test strips and non-invasive PAS. (**B**) The relationship between the measured blood glucose and the predicted glucose was evaluated using Clarke’s error grid [35]. Reprinted with permission from Ref. [35]. 2013, American Chemical Society.

The off-axis paraboloidal mirror is used to direct the laser beam toward the skin and focus it onto the contact surface. This creates a small spot on the skin where the laser is targeted. When the modulated laser hits the skin, the acoustic signal is generated and amplified by the T-shaped resonance cell; then it can be detected by the ultrasound microphone. After obtaining the PA signal, the data were analyzed and predicted by principal component analysis (PCA). Finally, the prediction data and the enzymatic test were compared, which is shown in Figure 7. The average estimation error determined from the correlation is approximately 11 mg/dL.

**Figure 8:**
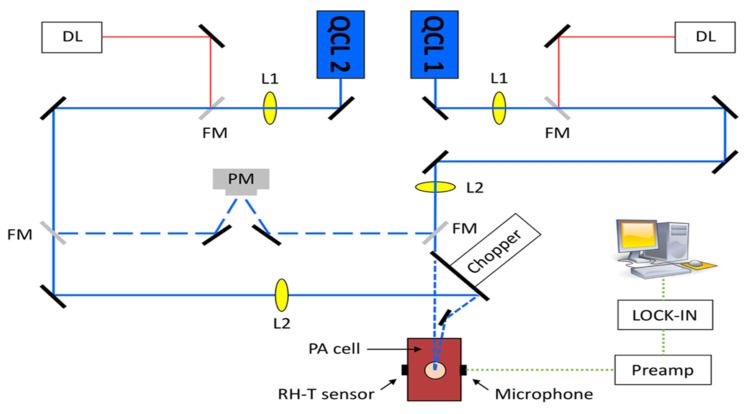
Experimental setup with two fixed-wavelength QCLs and the N_2_-ventilated PAC with free laser beam access [46]. Reprinted with permission from Ref. [46]. 2016, MDPI.

A different team of researchers also created a similar technique for in vivo applications [46]. The experimental arrangement for this setup is illustrated in Figure 8, where a dual-laser system has been utilized. The initial continuous wave external cavity quantum cascade laser (EC-QCL) used in the experiment can be tuned within a wavelength range of 1005 to 1080 cm^−1^, which includes two distinctive glucose absorption peaks at 1034 cm^−1^ and 1080 cm^−1^. Subsequently, a second pulsed EC-QCL was employed, with a tuning range of 1130 to 1310 cm^−1^. As a result, the first EC-QCL covered a range with strong glucose absorption, while the second EC-QCL contained a range where glucose absorption was either weak or negligible. Additionally, two QCLs with fixed wavelengths were selected, and their beams were directed alternately to the PAC by the flipping mirror (FM). Before reaching the PAC, both lasers were modulated by a mechanical chopper. Finally, the microphone attached to the setup was used to detect the photoacoustic signal.

**Figure 9:**
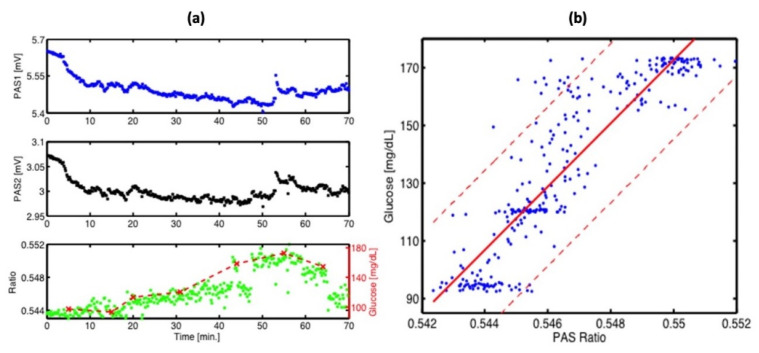
(**a**) Continuous PA signals at 1180 cm^−1^ (PAS1: blue) and 1080 cm^−1^ (PAS2: black) recorded at the fingertip. Correlation between the PA ratio signal (green) and simultaneous invasive blood glucose measurement (dashed red). (**b**) Correlation between invasive blood glucose data and non-invasive PA signals obtained from (**a**). The red solid line shows a linear fit (with R^2^ = 0.8) and the red dashed line represents the confidence bounds at a 90% confidence level [46]. Reprinted with permission from Ref. [46]. 2016, MDPI.

This experiment has two main improvements when compared to the previous studies. The first improvement is that the PAC was applied by a constant N_2_ flow during the experiment, which helped to reduce the relative humidity level to a stable level. The second improvement is that they utilized two fixed wavelength lasers selected for maximum and minimum glucose absorption, which can generate more reliable results. Figure 9 shows the experimental results, where a good correlation was observed. The new method of using two lasers can significantly enhance the stability of the measurement process, and a level of uncertainty of ±30 mg/dL in blood glucose concentration can be achieved with 90% confidence. However, this study did not use any data processing method, such as principal component analysis, and the outcomes were still inadequate for detecting glucose in the physiological range. To obtain a more accurate and stable result, more detection data and data manipulation techniques are needed.

In 2022, Aloraynan et al. used a single wavelength fixed at 1080 cm^−1^, which is the strongest absorption peak of glucose, and developed this technique by modulating the frequencies instead of the wavelength of the lasers. The usage of a single QCL was based on combining improvements from previous studies; the function generator was used to modulate the laser with square waves of 10 to 30 kHz and a duty cycle of 40%. Subsequently, the microphone, which had a maximum sensitivity between 15 and 30 kHz, was used to collect the PA signal.

**Figure 10:**
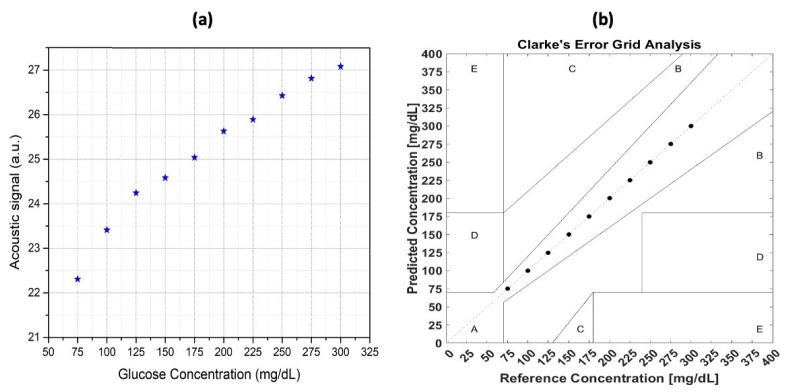
(**a**) The relationship between the glucose concentration of the samples and the photoacoustic signal was analyzed for correlation, and (**b**) the correlation between the sample glucose concentration and the photoacoustic signal after applying the machine-learning technique [63]. Reprinted with permission from Ref. [63]. 2022, MDPI.

The original raw data were collected and analyzed by integrating the area around the peak. A good correlation between the integral value and the glucose concentration was obtained, which is shown in Figure 10a. This result is satisfactory, but to be able to apply this technique in practice, a more accurate model is needed. However, it is very hard to improve the result from the perspective of the experimental setup. Hence, the data analysis technique is vital for improvement. Furthermore, a machine learning (ML) technique was employed in the data manipulation to obtain a more reliable and stable result, which is shown in Figure 10b.

ML is widely used in data analysis due to its satisfactory performance. A detailed review of the machine-learning techniques in glucose detection is introduced and discussed in the following section.

## 4. Machine Learning in Non-Invasive Glucose Detection

ML has recently been utilized in non-invasive glucose detection because of its outstanding performance in predicting the actual glucose level, which can help to improve detection sensitivity by using a complex mathematical model to correlate the collected data and the actual glucose level to meet FDA requirements. An ML model could be extremely helpful in accurately forecasting glucose levels both in vivo and in vitro, especially when dealing with various disrupting blood components (e.g., protein, urea, and cholesterol) and changes in environmental factors (such as temperature or humidity).

In general, non-invasive glucose detection can utilize both classification and regression techniques. The classification technique in ML predicts the class with a given input data [64]. The class, sometimes called labels or categories, is used to distinguish the different types of objects. In practical situations, the classification is used to predict the defined class, such as tumor detection and target marking. The predicted result is a definite and distinct label that represents a certain object. When it comes to glucose detection, the classification technique is frequently used to anticipate the discrete glucose concentration in vitro because the number of glucose samples is always finite and well-defined. Alternatively, the regression technique in ML is used to find the relationship between the independent input variables and the outcome. This technique can extract quantitative information, which has a continuous prediction value [63]. Furthermore, the regression technique can be utilized to forecast the continuous glucose concentration level in vivo, which has the capability to detect a wide range of glucose levels. In comparison, the classification technique can predict the glucose level more accurately, but lose some information in the intervals of the adjacent glucose level. Using the regression technique, it is possible to predict the glucose level across the entire range of desired glucose values. However, it cannot be used when the number of outcomes is finite.

As described earlier, there are different non-invasive methods in the literature to detect or quantify blood glucose levels. The capabilities of the ML model worked for basically every non-invasive glucose detection technique. In a word, the measurement result based on the different glucose levels was obtained many times, and each result had many features, depending on the chosen non-invasive method, which can correlate to the glucose level. The processing of the collected data set is summarized in Table 3.

According to Table 3, the features were mainly selected from the spectrum of the MIR light. Firstly, due to the extensive usage of prediction, regression was used as the major technique to predict the level of glucose at the early stage. To simplify the model and enhance computational efficiency, most researchers utilize principal component regression (PCR) to decrease the data’s dimensions. Moreover, partial least square regression (PLSR) was a stable, accurate, and robust model which is widely used in regression analysis. In recent times, classification techniques have been introduced to improve the model’s sensitivity and accuracy. Shokrekhodaei et al. employed various classification models, including support vector machine (SVM), decision trees (DT), and k-nearest neighbor (KNN), and found that the SVM model produced the highest accuracy with an F1 score of 0.99. Later on, an ensemble classification model (ECM) was used to further enhance the traditional classification model’s performance, and the specifics of this technique are explained in the following section.

ML is widely used today; the general process is described as follows. The whole dataset was first placed into training and testing set. Then, the reference value of the glucose level and the different features of the training set were added to the model and trained. Furthermore, after applying the model to the testing set, the prediction result was obtained. Accuracy is defined as how close the predicted value is to the reference value. If the selected features are highly correlated with the reference glucose value, the model’s performance is consistently satisfactory, as indicated by the Clarke error grid method.

As the purpose of this review is to give a comprehensive analysis of non-invasive blood glucose detection by MIR-based PAS, we confined the machine learning part to those topics. In general, in non-invasive glucose detection using optical methods, many researchers use the MIR absorption spectrum or the PA signal in different wave numbers as the features [66]. To improve the sensitivity of the PA signal, a resonance cell was used where the feature was extracted from a whole range of modulation frequencies [63]. Furthermore, other features (such as temperature, moisture, and personal characteristics) can be used to enhance the accuracy of the model.

### 4.1. Classification Methods

Classification techniques are capable of correlating with the entire range of glucose measurements of interest and can accurately predict hyperglycemia, normal, and hypoglycemia ranges of blood glucose levels [64]. Classification techniques handle each discrete glucose value separately without being affected by other glucose levels, which results in high prediction sensitivity. Some examples of the classification methods can be briefly described as K-nearest neighbor (KNN), decision tree (DT), and support vector machine (SVM). In the following section, an ensemble classification model is discussed and reviewed.

The ensemble classification model employs several learning algorithms to achieve superior predictive performance compared to the individual learning algorithms. This algorithm uses a random subspace sampling method for dividing the subgroups, which can extract random features and improve accuracy [67]. Then, every subspace will be trained independently with the validation dataset and perform the prediction. The classifier is one of the single classification models described above. In the end, this algorithm will choose the result that has the most votes among all the predicted results.

### 4.2. Regression Methods

Regression techniques can predict the continuous value of the glucose concentration. Then it can predict specific blood sugar levels more accurately than classification models. Furthermore, in real-time glucose monitoring systems, a regression can reflect the trends of the glucose in the time series, which is very useful for in vivo applications.

Some examples of regression models can be described as multiple linear regression (MLP), neural network (NN), partial least squares regression (PLSR), and principal component analysis (PCA). The regression methods are used in the in vivo environment. Then the reference data come from the oral glucose tolerance test (OGTT). We can use them to train the model with different methods to see the result and use RMSE to evaluate it.

Although ML algorithms have the potential to improve the clinical performance and accuracy of the conventional MIR-based PAS method, it requires many observations. The problem of overfitting [68,69,70] should also be carefully considered when building an ML-based calibration model using the above-mentioned methods.

## 5. Prospects of MIR-Based PAS

The proposed MIR-based PAS has the possibility to detect blood glucose non-invasively. However, the environmental parameters and interfering blood components should be considered and removed to attain a glucose monitoring system with good sensitivity, selectivity, and stability. The improvement for achieving such a system can be divided into two parts: (i) improvements of experimental methods for less interfering and stable signal, and (ii) analysis of the experimental data for a good correlation. In the following sections, we briefly discuss the future potential research directions for these two sections.

As stated, the absorption of water and other interfering components in the human blood are the main sources of noise for obtaining photoacoustic signals from glucose. First, the wavelength of the laser at the MIR wavelength region should be chosen in such a way that it is only sensitive to glucose to obtain a comparably good acoustic signal. To remove the background interfering components, another laser could be incorporated into the system to have a background (i.e., baseline) signal where it has a lower sensitivity to glucose. In our previous work [63], we used a 9.25 µm laser, which corresponds to the second absorption peak of glucose in the MIR region, as shown in Figure 3, to obtain the glucose signature from dummy samples. We achieved the detection limit of ± 25 mg/dL considering a single wavelength excitation source. Recently, we incorporated a second laser of 10.3 µm to improve the detection limit to ± 12.5 mg/dL with the other blood-interfering components [71]. Thus, a multiple wavelength-based MIR-PAS could be an ideal platform as it potentially removes the background and improves the signal-to-noise ratio, which in turn improves the overall sensitivity of the system.

Another strategy to remove the background signal is to use differential spectroscopy [23,58], where two laser wavelengths are chosen in such a way that the background signal from water and other components can be effectively removed. This technique was implemented in NIR-based PAS for non-invasive blood glucose detection [58]; however, the same strategies could also be implemented in the MIR region, which could lead to a more sensitive PAS system.

As discussed in the previous section, ML will play a significant role in analyzing and correlating the actual glucose level to the experimental results. In addition, due to the influence of the environment (temperature, pressure, humidity), the data obtained by the system will always have some outliers and fluctuations which affect the accuracy of the model. Then, the preprocessing of the data is needed to eliminate the impact. One way to achieve this is by removing the outliers [64]. Usually, the system will always give us a whole range of data. If we use all the original data as training features, we may lose some information that contributes more to the result. One way is to select the features that contribute most to the result. Then, we can use the Pearson correlation coefficient to extract the features which have the most relationship with the glucose concentration; this can greatly shorten the training time and attain a more reliable result.

There are different ML algorithms used for non-invasive glucose detection which could potentially improve the detection accuracy. However, this method particularly depends on the given dataset which may vary for different personnel. Thus, the problem of overfitting [68,69] should be considered when building a general ML model for MIR-based PAS.

Recently, Klonoff et al. identified seven analytical metrics for the evaluation of any non-invasive glucose monitoring system: bias, precision, effects of interfering substances, effects of physiologic states, effects of environmental or external conditions, sensor stability, and sources of variability (physiology, instrument, or environment) [70]. These factors must be considered and reported by any research group/company that wishes to claim any non-invasive glucose-sensing technology. Although MIR-based PAS has significantly improved over the last 10 years, to realize this technology as a potential non-invasive method with clinically approved accuracy, both experimental and data strategy techniques should be further improved.

## 6. Conclusions

In this review, we focused on the recent developments and progress of photoacoustic spectroscopy in the mid-infrared region for the detection of non-invasive blood glucose monitoring. This method is extremely promising for realizing future non-invasive techniques. Both the experimental procedures in MIR-based PAS and data analysis strategies were discussed that are published in the literature. Since photoacoustic signals can include various unwanted elements, it is essential to investigate them thoroughly to ensure that they are acquired correctly and produce precise outcomes. Therefore, it is imperative that the results obtained are precise. Although the published results in the literature have some limitations, several research prospects in this area could lead to the precise and non-invasive collection of the photoacoustic signal. The area of non-invasive blood glucose detection through MIR-based PAS is full of promise, but also presents challenges. In forthcoming research, deeper comprehension of photoacoustic signal generation, diverse biological samples’ data, and machine learning should enable scientists to tackle the aforementioned problems and guarantee the prosperous advancement of non-invasive blood glucose estimation technologies.

## Figures and Tables

**Table 1:** The normal range of blood glucose level of an adult human under different conditions was found through different test results [2].

Condition \Results	Fasting Blood Glucose Test (mg/dL)	Glucose Tolerance Test (mg/dL)	A1C Test (%)
Normal Pre-diabetic Diabetic	≤99	≤140	<5.7
	100–125	141–199	5.7–6.4
	≥126	≥200	≥6.5

**Table 2:** A detailed summary of the recent progress of non-invasive glucose sensing using MIR-based PAS. PAC: photoacoustic cell; QCL: quantum cascade laser; PA: photoacoustic; EC: external cavity.

Reference Year	MIR Sources	What Was Examined	PAC Used	Results
2005 Lilienfeld-Toal et al. [60]	Two QCLs—1080 cm^−1^ (9.26 µm) and 1066 cm^−1^ (9.38 µm)	The skin of the forearm	PAC with a twin measuring chamber, one for photoacoustic signal and another for background signal	The relationship between the PA signal produced and blood glucose levels are not likely to be very strong. However, the correlation was most significant when a 10 min time lag in blood glucose levels was considered.
2012 Kottmann et al. [42]	Tunable laser—1010–1095 cm^−1^ (i.e., 9.9–9.13 µm)	Human epidermal skin samples in contact with aqueous glucose solution	N_2_ ventilated PAC (77 mm^3^ volume) with gold coated on the inner surface and sealed with a diamond window	The PA signal is directly proportional to the concentration of glucose, both in a broad range of 0 to 10 g/dL and in a narrower range of 0 to 2000 mg/dL, compensating PA signal changes due to a variation of relative humidity and temperature. Detection limit—100 mg/dL.
2012 Pleitez et al. [35]	EC- tunable QCL—1000–1220 cm^−1^ (i.e., 10–8.2 µm)	An interstitial layer of the human skin	T-shaped PAC	Successfully measured in vivo glucose concentration from 50 mg/dL to 300 mg/dL and the measurement followed to the real blood glucose level without significant delay (<10 min).
2013 Pleitez et al. [57]	EC- tunable QCL—1000–1245 cm^−1^ (i.e., 10–8.03 µm)	Glucose in the human epidermis in the fingerprint region	Windowless PAC with two cylindrical cavities connected perpendicularly	The prediction value correlates well with the glucose concentration profile. The highest SNR of 72 was achieved at a resonance frequency of 51.7 kHz.
2013 Kottmann et al. [34]	Tunable QCL—1010–1095 cm^−1^ (i.e., 9.9–9.13 µm)	Glucose solutions with concentrations ranging from 0 to 5 g/dL; human skin at the fingertip and the forearm	Fiber-coupled PAC with a conically shaped PAC chamber and ventilated with N_2_.	In the entire range of 0–5 g/dL studied, the PA signal recorded showed a linear increase in glucose concentration (R^2^ = 0.993). An SNR of 1 was achieved with a detection limit of 57 mg/dL during in vitro testing. The signal generated at the fingertip was significantly stronger than that recorded at the forearm. However, the sensitivity obtained was not adequate for practical in vivo glucose detection.
2016 Kottmann et al. [46]	Two setups—(i) tunable QCLs, 1005–1100 cm^−1^ (i.e., 9.95–9.09 µm), and (ii) two lasers, 1080 cm^−1^ and 1180 cm^−1^ (i.e., 9.26 and 8.47 µm)	The skin of the human forearm	Fiber-coupled PAC	By using the dual-wavelength method, the stability of results was significantly enhanced, and the uncertainty in blood glucose concentration level was only 30 mg/dL at a 90% confidence level.
2018 Bauer et al. [61]	Tunable QCLs— 980–1245 cm^−1^ (i.e., 10.20–8.03 µm)	Index fingertip, thumb finger, hypothenar, and forearm	T-shaped PAC	PA detection errors were found to be higher than photothermal detection due to acoustic impedance mismatch. The index finger and thumb are the most suitable choices for glucose measurements because they have a dense network of capillaries, which facilitates the transfer of glucose from the blood to the ISF, and also because they have less fatty tissue.
2018 Sim et al. [62]	Tunable QCL—950–1240 cm^−1^ (i.e., 10.53–8.06 µm)	2D position scanning for the image at the fingertips	T-shaped PAC	Following careful hand washing, the valley between the ridges of the skin was found to be free from skin secretions, making it more resilient than the area where the eccrine sweat pores are situated on the top of the ridges. This discovery presents an encouraging prospect for achieving consistent results over consecutive days, as the skin’s exocrine activity and condition can be managed.
2022 Aloraynan et al. [63]	Single QCL—1080 cm^−1^ (9.26 µm)	Skin samples with different glucose concentrations	T-shaped PAC	The sensitivity of detection was improved to 25 mg/dL by employing a single wavelength QCL. The models created using unprocessed and processed data sets demonstrated a prediction accuracy of 86.7% and 90.4%, respectively.

**Table 3:** Machine-learning model used for non-invasive glucose detection in recent literature. PCR: principal component regression; PLSR: partial least square regression; MLR: multiple linear regression; KNN: k-nearest neighbor; DT: decision trees, SVM: support vector machine; FFNN: feedforward neural networks.

Date Reference	Selected Features	Model Used	Results
2013 Pleitez et al. [57]	1000 cm^−1^ to 1220 cm^−1^ MIR-PA signal	PCR, PLSR	The mean prediction error was found to be approximately 11 mg/dL.
2017 Kasahara et al. [65]	MIR absorption spectroscopy	MLR, PLSR	The maximum correlation coefficient of 0.49 was achieved.
2018 Sim et al. [62]	PA signal in the wavelength spectrum	PLSR, PCR	PCR and PLSR resulted in a Mean Absolute Relative Deviation of 8.95 and 8.67, respectively.
2018 Bauer et al. [61]	PA signal in the wavelength spectrum	PLSR	RMSE was cross-validated and standard deviations were obtained in the four different skin locations.
2021 Shokrekhodaei et al. [64]	Four wavelengths in the optical sensor	KNN, DT, SVM; MLR, FFNN	The FFNN model exhibits the smallest RMSE value of 11.1 mg/dL, whereas the SVM model demonstrates the highest F1 score of 0.99.
2022 Aloraynan et al. [63]	MIR-PA signal in 10–30 kHz	Ensemble Classification Model	Unprocessed and processed datasets achieved 86.7% and 90.4% prediction accuracy, respectively.

## Data Availability

Not applicable.

## References

[1] World Health Organization (2021). Global Report on Diabetes.

[2] American Diabetes Association, “Diabetes Tests”. https://www.cdc.gov/diabetes/basics/getting-tested.html.

[3] Geerlings S.E., Hoepelman A.I. (1999). Immune dysfunction in patients with diabetes mellitus (DM). FEMS Immunol. Med. Microbiol..

[4] Strategic Market Research (2021). Blood Glucose Monitoring Devices Market: By Product (Continuous Blood Glucose Monitoring Devices (Sensors, Transmitter & Receiver, Insulin Pumps), Self-Monitoring Devices (Blood Glucose Meter, Testing Strips, Lancets)), Applications (Type 1 Diabetes, Type 2 Diabetes), By End-User (Home Care, Diagnostics Centres, Hospitals), By Geography, Segment Revenue Estimation, Forecast, 2021–2030.

[5] Alsunaidi B., Althobaiti M., Tamal M., Albaker W., Al-Naib I. (2021). A Review of Non-Invasive Optical Systems for Continuous Blood Glucose Monitoring. Sensors.

[6] Delbeck S., Vahlsing T., Leonhardt S., Steiner G., Heise H.M. (2019). Non-invasive monitoring of blood glucose using optical methods for skin spectroscopy—Opportunities and recent advances. Anal. Bioanal. Chem..

[7] Huang J., Zhang Y., Wu J. (2020). Review of non-invasive continuous glucose monitoring based on impedance spectroscopy. Sens. Actuators A Phys..

[8] Tang L., Chang S.J., Chen C.-J., Liu J.-T. (2020). Non-Invasive Blood Glucose Monitoring Technology: A Review. Sensors.

[9] Hina A., Saadeh W. (2022). Noninvasive Blood Glucose Monitoring Systems Using Near-Infrared Technology—A Review. Sensors.

[10] Laha S., Rajput A., Laha S.S., Jadhav R. (2022). A Concise and Systematic Review on Non-Invasive Glucose Monitoring for Potential Diabetes Management. Biosensors.

[11] Nawaz A., Øhlckers P., Sælid S., Jacobsen M., Akram M.N. (2016). Review: Non-Invasive Continuous Blood Glucose Measurement Techniques. J. Bioinform. Diabetes.

[12] Shokrekhodaei M., Quinones S. (2020). Review of Non-Invasive Glucose Sensing Techniques: Optical, Electrical and Breath Acetone. Sensors.

[13] Buehler L.A., Balasubramanian V., Baskerville S., Bailey R., McCarthy K., Rippen M., Bena J.F., Lansang M.C. (2022). Noninvasive Glucose Monitor Using Dielectric Spectroscopy. Endocr. Pr..

[14] Sieg A., Guy R.H., Delgado-Charro M.B. (2004). Noninvasive Glucose Monitoring by Reverse Iontophoresis in Vivo: Application of the Internal Standard Concept. Clin. Chem..

[15] Tang F., Wang X., Wang D., Li J. (2008). Non-Invasive Glucose Measurement by Use of Metabolic Heat Conformation Method. Sensors.

[16] Kost J. (2002). Ultrasound-Assisted Insulin Delivery and Noninvasive Glucose Sensing. Diabetes Technol. Ther..

[17] Ballerstadt R., Evans C., Gowda A., McNichols R. (2006). In Vivo Performance Evaluation of a Transdermal Near- Infrared Fluorescence Resonance Energy Transfer Affinity Sensor for Continuous Glucose Monitoring. Diabetes Technol. Ther..

[18] March W., Lazzaro D., Rastogi S. (2006). Fluorescent Measurement in the Non-Invasive Contact Lens Glucose Sensor. Diabetes Technol. Ther..

[19] Esenaliev R.O., Larin K.V., Larina I.V., Motamedi M. (2001). Noninvasive monitoring of glucose concentration with optical coherence tomography. Opt. Lett..

[20] AEnejder A.M.K., Scecina T.G., Oh J., Hunter M., Shih W.-C., Sasic S., Horowitz G.L., Feld M.S. (2005). Raman spectroscopy for noninvasive glucose measurements. J. Biomed. Opt..

[21] Lambert J.L., Pelletier C.C., Borchert M. (2005). Glucose determination in human aqueous humor with Raman spectroscopy. J. Biomed. Opt..

[22] Malchoff C.D., Shoukri K., Landau J.I., Buchert J.M. (2002). A Novel Noninvasive Blood Glucose Monitor. Diabetes Care.

[23] Guo X., Mandelis A., Matvienko A., Sivagurunathan K., Zinman B. (2010). Wavelength-modulated differential laser photothermal radiometry for blood glucose measurements. J. Physics Conf. Ser..

[24] Marbach R., Koschinsky T., Gries F.A., Heise H.M. (1993). Noninvasive Blood Glucose Assay by Near-Infrared Diffuse Reflectance Spectroscopy of the Human Inner Lip. Appl. Spectrosc..

[25] Maruo K., Tsurugi M., Tamura M., Ozaki Y. (2003). In Vivo Noninvasive Measurement of Blood Glucose by Near-Infrared Diffuse-Reflectance Spectroscopy. Appl. Spectrosc..

[26] BMalik B.H., Coté G.L. (2010). Real-time, closed-loop dual-wavelength optical polarimetry for glucose monitoring. J. Biomed. Opt..

[27] Purvinis G., Cameron B.D., Altrogge D.M. (2011). Noninvasive Polarimetric-Based Glucose Monitoring: An in Vivo Study. J. Diabetes Sci. Technol..

[28] Vrančić C., Fomichova A., Gretz N., Herrmann C., Neudecker S., Pucci A., Petrich W. (2011). Continuous glucose monitoring by means of mid-infrared transmission laser spectroscopy in vitro. Analyst.

[29] Spanner G. (1996). New concept for the non-invasive determination of physiological glucose concentrations using modulated laser diodes. Anal. Bioanal. Chem..

[30] Kottmann J., Rey J.M., Sigrist M.W. (2011). New photoacoustic cell design for studying aqueous solutions and gels. Rev. Sci. Instrum..

[31] Spanner G., Niessner R. (1996). Noninvasive determination of blood constituents using an array of modulated laser diodes and a photoacoustic sensor head. Anal. Bioanal. Chem..

[32] Chen J., Furukawa H. (2023). Rapid and non-invasive detection of high-thickness glucose solution concentrations using quantum cascade laser-based transmission infrared spectroscopy. Infrared Phys. Technol..

[33] Pai P.P., Sanki P.K., Banerjee S. A photoacoustics based continuous non-invasive blood glucose monitoring system. Proceedings of the 2015 IEEE International Symposium on Medical Measurements and Applications (MeMeA).

[34] Kottmann J., Grob U., Rey J.M., Sigrist M.W. (2013). Mid-Infrared Fiber-Coupled Photoacoustic Sensor for Biomedical Applications. Sensors.

[35] Pleitez M.A., Lieblein T., Bauer A., Hertzberg O., von Lilienfeld-Toal H., Mäntele W. (2012). In Vivo Noninvasive Monitoring of Glucose Concentration in Human Epidermis by Mid-Infrared Pulsed Photoacoustic Spectroscopy. Anal. Chem..

[36] Rosencwaig A., Gersho A. (2008). Theory of the photoacoustic effect with solids. J. Appl. Phys..

[37] Zhang R., Luo Y., Jin H., Gao F., Zheng Y. (2020). Time-domain photoacoustic waveform analysis for glucose measurement. Analyst.

[38] Hazen K.H., Arnold M.A., Small G.W. (1998). Measurement of Glucose in Water with First-Overtone Near-Infrared Spectra. Appl. Spectrosc..

[39] Olesberg J.T., Arnold M.A., Mermelstein C., Schmitz J., Wagner J. (2005). Tunable Laser Diode System for Noninvasive Blood Glucose Measurements. Appl. Spectrosc..

[40] Khalil O.S. (2004). Non-Invasive Glucose Measurement Technologies: An Update from 1999 to the Dawn of the New Millennium. Diabetes Technol. Ther..

[41] Downing H.D., Williams D. (1975). Optical constants of water in the infrared. J. Geophys. Res. Atmos..

[42] JKottmann J., Rey J.M., Luginbühl J., Reichmann E., Sigrist M.W. (2012). Glucose sensing in human epidermis using mid-infrared photoacoustic detection. Biomed. Opt. Express.

[43] Gebhart S., Fowler R., Kapsner C., Lincoln D., McGee V., Pasqua J., Steed L., Wangsness M., Xu F., Vanstory M. (2003). Glucose Sensing in Transdermal Body Fluid Collected Under Continuous Vacuum Pressure Via Micropores in the Stratum Corneum. Diabetes Technol. Ther..

[44] Viengerov M.L. (1938). New method of gas analysis based on tyndall-roentgen optoacoustic effect. Dokl Akad Nauk SSSR.

[45] Kreuzer L.B. (2003). Ultralow Gas Concentration Infrared Absorption Spectroscopy. J. Appl. Phys..

[46] Kottmann J., Rey J.M., Sigrist M.W. (2016). Mid-Infrared Photoacoustic Detection of Glucose in Human Skin: Towards Non-Invasive Diagnostics. Sensors.

[47] GChristison G.B., MacKenzie H.A. (1993). Laser photoacoustic determination of physiological glucose concentrations in human whole blood. Med. Biol. Eng. Comput..

[48] Tuchin V.V. (2008). Handbook of Optical Sensing of Glucose in Biological Fluids and Tissues.

[49] Thennadil S.N., Rennert J.L., Wenzel B.J., Hazen K.H., Ruchti T.L., Block M.B. (2001). Comparison of Glucose Concentration in Interstitial Fluid, and Capillary and Venous Blood During Rapid Changes in Blood Glucose Levels. Diabetes Technol. Ther..

[50] Liakat S., Bors K.A., Huang T.-Y., Michel A.P.M., Zanghi E., Gmachl C.F. (2013). In vitro measurements of physiological glucose concentrations in biological fluids using mid-infrared light. Biomed. Opt. Express.

[51] Wang L.V., Wu H. (2012). Biomedical Optics: Principles and Imaging.

[52] Rosencwaig A. (1973). Photoacoustic Spectroscopy of Biological Materials. Science.

[53] Mackenzie H.A., Ashton H.S., Spiers S., Shen Y., Freeborn S.S., Hannigan J., Lindberg J., Rae P. (1999). Advances in Photoacoustic Noninvasive Glucose Testing. Clin. Chem..

[54] Hugi A., Terazzi R., Bonetti Y., Wittmann A., Fischer M., Beck M., Faist J., Gini E. (2009). External cavity quantum cascade laser tunable from 7.6 to 11.4 μm. Appl. Phys. Lett..

[55] Rassel S., Xu C., Zhang S., Ban D. (2020). Noninvasive blood glucose detection using a quantum cascade laser. Analyst.

[56] El-Busaidy S., Baumann B., Wolff M., Duggen L., Bruhns H. (2019). Experimental and Numerical Investigation of a Photoacoustic Resonator for Solid Samples: Towards a Non-Invasive Glucose Sensor. Sensors.

[57] Pleitez M.A., Lieblein T., Bauer A., Hertzberg O., Von Lilienfeld-Toal H., Mantele W. (2013). Windowless ultrasound photoacoustic cell forin vivomid-IR spectroscopy of human epidermis: Low interference by changes of air pressure, temperature, and humidity caused by skin contact opens the possibility for a non-invasive monitoring of glucose in the interstitial fluid. Rev. Sci. Instrum..

[58] Tanaka Y., Tajima T., Seyama M., Waki K. (2020). Differential Continuous Wave Photoacoustic Spectroscopy for Non-Invasive Glucose Monitoring. IEEE Sens. J..

[59] Guo X., Mandelis A., Zinman B. (2012). Noninvasive glucose detection in human skin using wavelength modulated differential laser photothermal radiometry. Biomed. Opt. Express.

[60] von Lilienfeld-Toal H., Weidenmüller M., Xhelaj A., Mäntele W. (2005). A novel approach to non-invasive glucose measurement by mid-infrared spectroscopy: The combination of quantum cascade lasers (QCL) and photoacoustic detection. Vib. Spectrosc..

[61] ABauer A., Hertzberg O., Küderle A., Strobel D., Pleitez M.A., Mäntele W. (2018). IR-spectroscopy of skin in vivo: Optimal skin sites and properties for non-invasive glucose measurement by photoacoustic and photothermal spectroscopy. J. Biophotonics.

[62] JSim J.Y., Ahn C.-G., Jeong E.-J., Kim B.K. (2018). In vivo Microscopic Photoacoustic Spectroscopy for Non-Invasive Glucose Monitoring Invulnerable to Skin Secretion Products. Sci. Rep..

[63] Aloraynan A., Rassel S., Xu C., Ban D. (2022). A Single Wavelength Mid-Infrared Photoacoustic Spectroscopy for Noninvasive Glucose Detection Using Machine Learning. Biosensors.

[64] Shokrekhodaei M., Cistola D.P., Roberts R.C., Quinones S. (2021). Non-Invasive Glucose Monitoring Using Optical Sensor and Machine Learning Techniques for Diabetes Applications. IEEE Access.

[65] RKasahara R., Kino S., Soyama S., Matsuura Y. (2018). Noninvasive glucose monitoring using mid-infrared absorption spectroscopy based on a few wavenumbers. Biomed. Opt. Express.

[66] Sankhala D., Sardesai A.U., Pali M., Lin K.-C., Jagannath B., Muthukumar S., Prasad S. (2022). A machine learning-based on-demand sweat glucose reporting platform. Sci. Rep..

[67] Ho T.K. (1998). The random subspace method for constructing decision forests. IEEE Trans. Pattern Anal. Mach. Intell..

[68] Zech J.R., Badgeley M.A., Liu M., Costa A.B., Titano J.J., Oermann E.K. (2018). Variable generalization performance of a deep learning model to detect pneumonia in chest radiographs: A cross-sectional study. PLoS Med..

[69] Rodríguez-Rodríguez I., Chatzigiannakis I., Rodríguez J.-V., Maranghi M., Gentili M., Zamora-Izquierdo M.-Á. (2019). Utility of Big Data in Predicting Short-Term Blood Glucose Levels in Type 1 Diabetes Mellitus Through Machine Learning Techniques. Sensors.

[70] Klonoff D.C., Nguyen K.T., Xu N.Y., Arnold M.A. (2021). Noninvasive Glucose Monitoring: In God We Trust—All Others Bring Data. J. Diabetes Sci. Technol..

[71] Aloraynan A., Rassel S., Kaysir R., Ban D. (2023). Dual quantum cascade lasers for noninvasive glucose detection using photoacoustic spectroscopy. Sci. Rep..

